# Learning from the Cardiologists and Developing Eluting Stents Targeting the Mtor Pathway for Pulmonary Application; A Future Concept for Tracheal Stenosis

**DOI:** 10.4172/1747-0862.1000065

**Published:** 2013-08-26

**Authors:** Paul Zarogoulidis, Kaid Darwiche, Kosmas Tsakiridis, Helmut Teschler, Lonny Yarmus, Konstantinos Zarogoulidis, Lutz Freitag

**Affiliations:** 1 Pulmonary Department-Oncology Unit, “G. Papanikolaou” General Hospital, Aristotle University of Thessaloniki, Thessaloniki, Greece; 2 Department of Interventional Pneumology, Ruhrlandklinik, West German Lung Center, University Hospital, University Duisburg-Essen, Essen, Germany; 3 Cardiothoracic Surgery Department, “Saint Luke” Private Hospital of Health Excellence, Panorama, Thessaloniki, Greece; 4 Pulmonary Department, Ruhrlandklinik, West German Lung Center, University Hospital, University Duisburg-Essen, Essen, Germany; 5 Division of Pulmonary and Critical Care Medicine, Sheikh Zayed Cardiovascular & Critical Care Tower, Johns Hopkins University, Baltimore, USA

**Keywords:** mTOR, Stents, Stenosis

## Abstract

Tracheal stenosis due to either benign or malignant disease is a situation that the pulmonary physicians and thoracic surgeons have to cope in their everyday clinical practice. In the case where tracheal stenosis is caused due to malignancy mini-interventional interventions with laser, apc, cryoprobe, balloon dilation or with combination of more than one equipment and technique can be used. On the other hand, in the case of a benign disease such as; tracheomalacia the clinician can immediately upon diagnosis proceed to the stent placement. In both situations however; it has been observed that the stents induce formation of granuloma tissue in both or one end of the stent. Therefore a frequent evaluation of the patient is necessary, taking also into account the nature of the primary disease. Evaluation methodologies identifying different types and extent of the trachea stenosis have been previously published. However; we still do not have an effective adjuvant therapy to prevent granuloma tissue formation or prolong already treated granuloma lesions. There have been proposed many mechanisms which induce the abnormal growth of the local tissue, such as; local pressure, local stress, inflammation and vascular endothelial growth factor overexpression. Immunomodulatory agents inhibiting the mTOR pathway are capable of inhibiting the inflammatory cascade locally. In the current mini-review we will try to present the current knowledge of drug eluting stents inhibiting the mTOR pathway and propose a future application of these stents as a local anti-proliferative treatment.

## Introduction

There are either benign or malignant cases where stent placement is necessary [1-5]. There have been proposed several classification systems according to the type and length of stenosis [6]. In addition, only experienced pulmonary physicians with continuous and systematic training in interventional pulmonary medicine should perform these interventional techniques [7]. In the case of benign disease such as; tracheomalacia or airway fistula introducing a stent is indicated upon diagnosis [8,9]. Respiratory failure is observed in tracheomalacia and therefore stent insertion is necessary [10]. There is also the case where airway and esophagus fistulas are present at the same time [9]. On the other hand in the case of malignancy usually debulking of the tumor tissue is necessary prior to the stent placement. The interventional treatment can be performed either as minimal invasive without deep sedation or using the rigid bronchoscope with the help of anesthesiologists [11,12]. The pulmonary physician has many arrows in the quiver from which he can choose to use based on the shape, location, tissue and of course experience. There are several probes such as; apc, laser, electro-knife, cryo and loop [3]. However; formation of granuloma tissue can occur at both ends of the stent in both situations due to local tissue stress, and local tissue hypoxia which activates an inflammatory cascade releasing cytokines and chemokines [13]. Moreover, the vascular endothelial factor (VEGF) is locally overexpressed further augmenting the abnormal tissue formation [14]. There has been also reported the case where relapsing stenosis was observed in patients after cyclophosphamide treatment [1]. Therefore patients should be regularly observed as outpatients since there is no specific time where the abnormal tissue formation begins and no specific cell time proliferation. Local interaction between the stent and the factors previously presented are not influencing the tissue formation in the same manner in all patients. Currently interventional pulmonology uses the previously presented apparatus plus balloon dilation [15]. There are currently several stents on the market each one with advantages and disadvantages [16-18]. Furthermore, different techniques for stent insertion have been proposed and there is long term experience with almost all stents regarding adverse event records [19,20]. Several adjuvant therapies to prevent tracheal stenosis or prolong the granuloma tissue reformation have been used such as; mitomycin C and local steroid injection, however; more efficient local treatments are in demand [15]. Learning from the experience of the cardiothoracic surgeons currently there are eluting stents targeting the mammalian target of rapamycin (mTOR) pathway with sirolimus (SES), everolimus (EES), zotarolimus (ZES) and paclitaxel (PES) [21-23]. These stents have local immunomodulatory and anti-inflammatory effects by regulating locally several homestatic mechanisms and block abnormal vascularigenesis [24-26]. Furthermore, the existing stents have been investigated with different carriers as a delivery system in order to prolong the drug administration from the stent. The carrier used can be summarized to: i) novel abluminal groove-filled biodegradable polymer [27], ii) polymer-free phospholipid encapsulated [28 ], iii) Nanoporous CREG-eluting stent [29], iv) novel fully bioabsorable poly-L-lactic acid [30], v) absorb everolimus eluting bioresorbablevascular scaffolds in small vessels [31]. Therefore they present a future application for airway stent, because apart from their ability to sustain the structure of the airways they will prevent abnormal granuloma tissue formation or prolong already treated granuloma lesions. In the current mini-review we will present the current knowledge on eluting stents for angioplasty usage and we will propose a future treatment methodology with eluting stents for airway application.

## Mtor Pathway and Inhibitory Drugs

**Figure 1:**
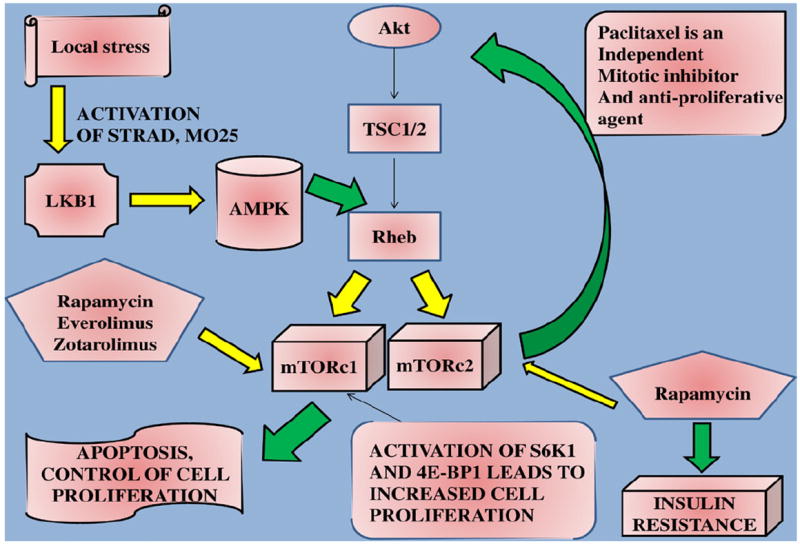
**LKB1;** liver kinase B1, **TSC1/2;** tuberous sclerosis complex, **mTORc1/2;** mammalian target of rapamycin complex 1-2, **STRAD;** Ste20-like adaptor protein, **MO25;** Mouse protein 25 alpha, **S6K1;** Ribosomal protein S6 kinase beta-1,**4E-BP1;** Eukaryotic translation initiation factor 4E-binding protein 1,**Rheb;** Ras homolog enriched in brain, **Akt;** Protein Kinase B (PKB), **AMPK;** adenosine mono-phosphate-activated protein kinase.

The mammalian target of rapamycin (mTOR) pathway is involved in the cell function by evaluating the energy status, integrate signals from circulating factors and finally regulate several homestatic systems, such as; ribosome biogenesis and autophagy. It is actually a serine-threonine kinase enzyme complex (290 kDa) [24]. The complex production and involvement in the metabolism is associated with stress, cellular growth, phosphatidylinositol 3-kinases (PI3K)-Akt-dependent mechanisms and vascularigenesis [25]. Only a single mTOR gene is located in the 1p36.2 chromosome and it consists of; a) mTORC_1_; consists of G protein beta subunit-*like* and the regulatory-associated protein of mTOR (raptor) and b) mTORC_2_; consists of G protein beta subunit-*like* and rapamycin intensive component of mTOR (rictor). Sirolimus (rapamycin) inhibits only mTORC_2_. Rapamycin targets both mTORC_1_ and mTORC_2_, on the other hand everolimus targets only mTORC_1_. Zotarolimus targets both mTORC_1_ and the cyclin-dependent kinases such as cdk2. Paclitaxel block the cell division in the G-phase (Figure 1).

Rapamycin is a macrolide and immunosuppressant that is used against organ rejection [32]. It inhibits the T-cell and B-cell response to interleukin-2 (IL-2). The rapamycin molecules bind to the cytosolic protein *FK-binding protein 12* (FKBP12) and subsequently directly inhibit the mTOR Complex 1. Sirolimus eluting stents (SES) are certified and on the market as anti-proliferative agents against restenosis of coronary vessels [33]. However; there are data indicating that these stents may increase the risk of thrombosis [34], clinical data will be presented below in the sirolimus studies section. Sirolimus and mTOR inhibitors have been accused of causing hypersensitivity pneumonitis alone or in combination with other drugs [35-37]. These situations were observed to be increased in patients with underlying respiratory disease [38,39]. However; the carrier of the stents play a major role as in the case of polymers which have been identified as a drug molecule that induces hypersensitivity pneumonitis [40]. Moreover; diabetes like syndrome might occur with insulin insensitivity and glucose intolerance due to the disruption of mTOR Complex 2 [41]. Sirolimus has been also identified to lower the risk of cancer in organ transplant patients [42]. Additionally, if dosed appropriately the immune response against malignancy is enhanced [43].

Everolimus is a derivate of sirolimus and it is used as an immunosuppressant for organ transplant [44] and against several types of cancer [45-48]. It is effective only against the mTORC_1_ protein and not the mTORC_2_. Via inhibition of mTORC_1_ hyper-activation of the protein kinase B (PKB) is occurred. Currently the drug is certified as a coating for coronary stents as an anti-proliferative agent.

Zotarolimus is a semi-derivative of rapamycin and it has been designed for encapsulation in phosphorylcholine carriers for coronary stent application. The zotarolimus stent anti-proliferative effect has been tested in more than 120.000 patients [49,50]. The zotarolimus and rapamycin have the advantage of blocking cell cycle and induce apoptosis by inhibiting the cyclin-dependent kinases such as cdk2. Therefore zotarolimus and rapamycin are more efficient at least based on their pharmacological activity as they block with multiple ways the mTOR regulating pathway and induce more extensive apoptosis. However; hypersensitivity pneumonitis has been reported with zotarolimus eluting stents [51] and catastrophic multi vessel spasms [52]. It should be mentioned that the pneumonitis was finally attributed to the polymer carrier and in the second case the spasms were resolved and the patient was discharged.

Paclitaxel is mitotic inhibitor and it is used for cancer treatment (lung, breast, ovarian and Kaposi) and for restenosis treatment (paclitaxel eluting stents). It can be found in the market dissolved in Cremophor EL^®^ and ethanolor albumin. It belongs to the taxane family (docetaxel and paclitaxel) and it stabilizes the microtubules during cell division [53]. Paclitaxel does not inhibit microtubule assembly like colchicine, but stabilizes the microtubule polymer. The progression of mitosis is blocked and apoptosis or reversion of the G-phase is observed [54]. Paclitaxel is a certified drug as a coating anti-proliferative agent for coronary stents and it has been approved for coronary restenosis [55].

## Sirolimus

Sirolimus stents have been widely used in many studies and with many new nanocarriers in order to identify a tissue friendly coating material and to enhance the prolonged local drug release effect [27-30,56-58]. In the study by Kozuma et al. [57] data were pooled from the RESTART (Japanese registry for patients) (611pts) that had SES stents implanted and they were evaluated with coronary angiograms. The follow-up was for 12-months and the patients were divided into three groups the early-ST (EST) events, late-ST (LST) events and very late-ST (VLST) events. The analysis demonstrated that residual dissection was more frequent in the EST group. Peri-stent contrast staining and stent fracture were observed to be increased in the VLST group *p*<0.001 and *p*<0.001 respectively. Moreover; incidence and predictive factors for late target lesion vascularization (TLR) have been identified in 249 patients with SES implantation. These patients had a 5-year follow-up and the TLR incidence was 2.1% per year. There were three major factors associated with TLR; i) young age (*p*=0.026), ii) stent fracture (*p*=0.012) and iii) insulin treated diabetes mellitus (*p*=0.001) [59]. In a large meta-analysis study by Luca et al. [60] data comparing FG-DES to BMS in ST-segment elevation myocardial infarction (STEMI) patients were presented, and again it was observed that SES and PES stents reduce the TVR in patients with diabetes. A further evaluation of the cost-effectiveness of SES and BMS implantation in diabetic and non-diabetic patients indicated that currently both stent types are equally priced [61]. Patients with diabetes are commonly diagnosed with coronary disease and are a major group of patients receiving stents. The SYNTAX score is an angiographic tool indicated to the clinician the optimal revascularization technique in patients with left main and/or three-vessel disease [62,63]. The Clinical SYNTAX (CSS) was evaluated by the HARA et al. [64] group which additionally combines age, serum creatinine and ejection fraction. The new proposed system CSS predicts long-term outcomes in patients receiving SES stents more efficiently in comparison to the SYNTAX score. In the PRISON II study 200 patients were enrolled and BMS stents were evaluated versus SES for in-stent very late luminal loss (VLLL) and additional late luminal loss (ALLL) at five years with angiography [58]. The 5-year follow-up major results indicated that the in-stent VLLL was lower in the SES group than in the BMS group (*p*=0.09), however, the in-segment VLLL was similar with both stents (*p*=0.89). The SES stents were compared to EES in a sub-group of patients from the RESET trial in 571 patients with a 12 month follow-up. The major results indicated that the EES stents are superior to SES as less stent fractures and less peri-stent staining was observed in the EES group (*p*=0.18). However; late loss of the proximal edges was observed to be lower in the SES stents (*p*=0.05) [65]. The EES stents were evaluated in comparison to SES and PES in 1.481 patients with acute coronary infarction and after a 2 year follow-up the MACE rate was observed to be lower with then (*p*=0.02). The stent thrombosis events were also observed to be lower with EES stents in comparison to SES or PES (*p*=0.16)[66]. In the study by Mischie et al. [67] a head to head comparison was performed between SES and BMS in 48 patients. In the same patient with multiple lesions both SES and BMS were implanted. The endothelial dysfunction was observed by measuring vessel diameter variation before and after implantation. The major results indicated that both vasoconstriction and endothelial dysfunction were increased in the SES stents. Furthermore; in a seven year follow-up of 434 patients which had either BMS or SES stents implanted, it was observed that there was no significant difference for MACE events [68]. The clinical outcomes were in favor of the patients with SES for the first year, however; in the long-term follow-up there was no superiority observed between SES and BMS. In another study SES implantation proximal to a BMS implantation in the same vessel in patients with ischemic heart disease inhibited neointimal proliferation in the BMS stents (*p*<0.005) [69]. However; in another case where two SES stents were implanted the one close to the other inside the same vessel lumen, a membranous diaphragm formation was observed with optical coherence tomography after one year [70]. Novel SES stents with different carriers as a coating have been investigated. In the study by Qiu et al. [30] a novel fully bioabsorable poly-L-lactic acid sirolimus-eluting stent was applied in 12 minipigs without any complications. Neointimal hyperplasia was prevented for 28 days and in only one stent 50% lumen reduction was observed. In another study by Lemos et al. [28] a novel polymer-free phospholipid encapsulated SES was constructed and evaluated in rabbits. There were two forms: i) stent-plus-balloon and ii) stand-alone-balloon catheter. The evaluation was performed with: i) inflammation score, ii) fibrin score, iii) Schwartz injury score and iv) Gunn injury score. Reduced neointimal hyperplasia was observed with low systemic drug release and this was the first study reporting eluting drug from a stainless platform. The drug penetrated efficiently all the layers of the vessels. The novel FIREHAWK^®^ SES stent was also investigated and compared to EES in 458 patients [27]. The following parameters were investigated and they were found to be similar in both groups: i) ischaemia-driven target lesion revascularization, ii) target vessel myocardial infarction, iii) target lesion failure, iv) in-stent late lumen loss. In the study by Christiansen et al. [56] two different SES stents were investigated and compared in 2468 patients: i) biolimus-eluting biodegradable polymer-coated SES stent versus the ii) durable polymer-coated SES. The stent thrombosis rate was increased in the patients that received the biolimus stent (*p*=0.034). The clinical results indicated that the inferiority of the biolimus stent, however; long term follow-up of these patients (>12 months) with provide additional data. Finally, in the study by Deng et al. [29] the nanoporous cell-specific pharmacokinetic effect stent (CREG) was investigated and compared to SES and BMS *in vitro* and *in vivo*. The CREG and SES inhibited in the same degree the *in vitro* vascular smooth muscle cell proliferation. However; the human endothelial cell proliferation was inhibited only by SES and increased by CREG stents. The neointimal formation was attenuated for four weeks in comparison to BMS and the inhibition among the three stent types was observed as follows: SES>CREGES>BMS. Increased reendothelialization was observed in the CREGES stent group compared to the SES and BMS (Table 1).

## Everolimus

In the review study by Park et al. an extended search was performed in order to identify the differences regarding safety and efficacy of everolimus eluting stents (EES) versus the sirolimus eluting stents (SES). The systematic review and analysis of the data indicated that with the EES the trend of stent thrombosis and repeat revascularization are lowered significantly. Additionally, less myocardial infarction events were observed with the EES [71]. Long-term application of second generation EES (SG-EES) has been also compared to the first generation drug eluting-SES in small vessels [72]. After 1 year follow up major adverse cardiovascular effects were observed 9.1% in second generation-EES and 8.6% in SES, however; 0% thrombotic events were observed in EES and 1.2% in SES. It has to be mentioned that more systemic hypertension was seen in the EES group. Moreover; the same concept was applied in patients with total coronary occlusion with 1 year follow-up, these patients are in high risk of restenosis and revascularization. In the EES group less restenosis 9.1% vs. 10.8% and less MACE for EES 11.1% vs. 15.9% for SES were observed [73]. Neointimal hyperplasia and peri-stent arterial remodeling after EES and SES implantation was evaluated with intravascular ultrasound (IVUS). In the EES group less relative change in the vessel and less plaque volume index was observed (*p*=0.030 and *p*=0.016). Moreover; less late acquired stent malapposition (LASM) and positive peri-stent vascular remodeling defined as an increase in vessel volume index>10% was observed (*p*<0.001 and *p*=0.027) [74]. The SG-EES were also compared in a larger study with 2.126 and with a two year follow up vs. first generation eluting stents (SES) and (PES). The SG-EES vs. FG-SES demonstrated lower rate of target vessel revascularization (TVR) and lower stent thrombosis (ST) thrombosis. Whereas in the second group of SG-EES vs. FG-PES less major adverse cardiovascular events were observed (MACE) meaning including death events and myocardial infarction events. In both groups no ST was observed in the EES after the first 3 months [75]. The same positive results were observed with 317 patients again with EES and FG-DESs in another study applying these stents in saphenous vein graft lesions [76]. Less events of target lesion revascularization, target vessel revascularization, MACE and ST were observed. Evolution in nano-materials and further investigation of novel nano-carriers improved the local interaction between tissue and stent surface by enhancing the bioavailability [77]. In the study by Smits et al. [77]abluminal biodegradable polymer biolimus-eluting stentsvs. durable polymer everolimus-eluting stents were evaluated for safety and efficacy and biodegradable polymer biolimus-eluting stents were found to have no significant differences regarding the trend of MACE. Therefore longer follow up (>1 year) is necessary for further evaluation (Table 1).

## Zotarolimus

In the study by Park et al. [78] second generation stent of EES and ZES were compared in 5.054 patients and both stents had similar ST rates (*p*=0.306). Moreover; the patient related outcome (*p*=0.702) and stent-related outcome (*p*=0.662). The strongest predictor for target lesion failure was off label application of a stent (*p*=0.015). Both stents after one year of follow-up demonstrated similar results. Again in the Twente study with 1.391 patients enrolled target vessel revascularization, target vessel failure, target vessel-related myocardial infarction and composite of cardiac death values did not differ among the two groups (*p*=0.65) after 2 years of follow-up [79]. The ST events were 1.2% for ZES and 1.4% for EES (*p*=0.63). In another study 60 patients had either EES or ZES stent placement and had their neointimal coverage and malapposition evaluated at 3 month and 12 month with coherence tomography [80]. The neointimal hyperplasia was observed to be higher at 3 months in ZES stents, however; at 12 months the patients with EES stents had better vascular healing. No significant number of ST events was observed between the two groups. The ZES stents have been compared to PES in a trial with 400 patients enrolled and it was observed that in-stent late lumen loss (LLL) was less in ZES stents (*p*<0.001). Target lesion revascularization was 1.5% for ZES and 7% for PES (*p*=0.011). The target lesion failure was 5.6% for ZES and 11% for PES [81]. Moreover; in patients with bifurcated lesions SES or EES were firstly implanted and afterwards ZES were implanted [82]. The lesions were evaluated with a 3-dimensional quantitative coronary analysis software and less “side-branch” trouble was observed when compared to ZES 4% and SES 16% (*p*=0.014) and EES 11% (*p*=0.12). Additionally, at the end of the procedure the minimal-lumen-diameter at the side-branch point was larger in the ZES group compared to EES and SES (*p*=0.008). In the study by Van den Branden et al. [83] the in-segment and in-late stenosis were evaluated with Endeavor ZES/Resolute ZES and SES. The SES group presented higher angiographic outcomes compared to the Endeavor ZES, however; SES and Resolute ZES comparison presented similar results. Endeavor zotarolimus eluting stents were compared with first generation drug eluting stents and bare-metal stents (BMS) in 3.616 patients [84]. There were less target lesion revascularization with E-ZES compared to BMS (*p*<0.001), however; the rate was similar when compared to FG-DES (*p*=0.63). Mace events were also lower with E-ZES vs. FG-DES and BMS. It should be mentioned that TLR rate was high in the first year of implantation for E-ZES, but lowered significantly afterwards in the 5 year follow-up and increased for FG-DES. Finally, two major adverse events have to be reported: i) repeated catastrophic multi-vessel coronary spasm was observed after E-ZES implantation, the patient was discharged after treatment [52] and ii) hypersensitivity pneumonitis from ZES [51] which was attributed to the polymer coating of the stent as reported in the Research on Adverse Drug Events and Reports (RADAR project) [40] (Table 1).

## Paclitaxel

Paclitaxel eluting stents (PES) were compared to bare metal stents (BMS) and percutaneous transluminal angioplasty (PTA) in a 2 year study with 787 patients [85]. The PES when compared to the control group had a 2 year event free survival (*p*=0.02) and higher clinical benefit (*p*=0.05) when compared to BMS group. A long term superiority for PES compared to PTA and provisional BMS was observed. In another study data were pooled from the Cardiovascular Atherosclerosis and Percutaneous Transluminal Interventions (CAPTAIN) registry in total 420 patients with ostial lesions were treated with CYPHE, TAXUS or BMS [86]. In the BMS group higher late loss (*p*=0.006) and restenosis rate (*p*<0.001) compared to the CTPHER and TAXUS stents was observed. However; the BMS in the long term follow-up had higher target lesion revascularization than the CYPHER and TAXUS (*p*=0.002). The cardiac event-free survival rate was also lower in the BMS group than in the CYPHER and TAXUS (*p*<0.001). A head-to-head comparison of PES vs. SES was performed by the group of Erdim R. et al. [87] in 127 patients with ST segment elevation myocardial infarction (STEMI). The MACE events were 8.3% for SES and 16.4 % for PES (*p*=0.28). Rates for early ST versus late ST for SES vs. PES differ (*p*>0.005) being more increased in the PES group. However; in the two year follow-up there was no statistical significance between the two types of stents. In a study evaluating the PES vs. EES stents in the left main coronary artery for 3 years it was observed that treatment with two stents was more frequent in patients treated with PES [23]. The three year definite and probable thrombosis rate was 1.6% for PES and 1.4% for EES (*p*=0.80). Target lesion revascularization was 83.6% PES versus 82% EES (*p*=0.60) and the 3 year death and infarction survival rates were 86.1% for PES and 87.3% for EES (*p*=0.50). Six year long term follow-up was also investigated for PES and SES and no significant differences were observed for MACE between the two stent types (*p*=0.52) and TLR (*p*=0.68) [88]. Additionally, it was observed that the stent type was not a predictive factor for MACE (*p*=0.87) or TLR (*p*=0.38). The new PES stent Coroflex Please (B Braun, Melsungen, Germany) was compared to TAXUS in 945 patients, however; it was found to be inferior based on clinical and angiographic findings [89]. In specific ST rate was higher in Coroflex vs. Taxus (*p*=0.317) and also myocardial infarction events were higher in the Coroflex group (*p*=0.12). Moreover; Bivalirubin and PES were evaluated in patients undergoing primary percutaneous coronary intervention (PCI) of the left anterior descending artery (LAD) vs. non-LAD patients (3.32pts) [90]. MACE events were higher in the LAD PCI patients (*p*=0.013) and cardiac deaths were significantly increased (*p*=0.001). Patients receiving bivalirubin vs. unfractioned heparin plus glycoprotein IIb/IIIa inhibitor had lower MACE events (bivalirubin group). Additionally, patients with PES stents had reduced revascularization rates in LAD patients, which are the patients with increased risk for cardiovascular events. A new paclitaxel-coated poly-L-lactide acid (PLLA) biodegradable biopolymer stent has been constructed and investigated in dogs for a benign biliary stricture application. Different coatings were investigated and it was observed that the granulation tissue formation was efficiently controlled locally [91] (Table 1).

## Conclusions

The main issue that has to be dealt either in malignant or benign stenosis is the application of an efficient and prolonged local treatment. In the case of malignancy the underlying disease should be treated at the same time locally and systematically. There are currently several certified and experimental local treatments for airway malignancy [3,7,12,92-101]. After local treatment in the case of malignancy and stent placement or diagnosis of benign disease and stent placement follow-up of the patient is necessary for granuloma tissue formation. In both cases the formation of granuloma tissue can occur in the former normal tissue. We presented in the current mini-review the data from 4 different drug eluting stent types that are on the market with certified approval to be used in coronary diseases. We did not include the bare metal stents as these are considered inferior to the immunomodulatory and anti-inflammatory ability of the SES, EES, ZES and PES stents. The sirolimus, everolimus and zotarolimus belong in the macrolide family and their immunomodulatory and anti-inflammatory ability in the respiratory system is well known [102]. Paclitaxel is also a known mitotic inhibitor which can regulate locally the abnormal cell proliferation [53]. Rapamycin (SES) suppresses the endothelial proliferation and migration through down-regulation of miR-2 [26]. In addition the BMS coated or uncoated are already in the market for pulmonary use and we have data regarding their performance and interaction with the airway tissue. The major drawback of the existing stents for coronary disease is their short drug eluting time which rarely is prolonged more than 2 months at least for those types that have been approved and already on the market. After the stents become inactive and do not elute locally any drug it has been observed that they tent to cause in-stent thrombosis and of course they will be always “a foreign” material in the patients’ body. Therefore we would like to have with current technology a material that is fully bioabsorbable and a carrier that penetrates all layers of the airways. However; further experimentation is necessary since the stent is also a local method for maintaining the structure of the airways and local tool of support. There are also major differences between the two systems of application. The respiratory system has local defense mechanisms throughout the respiratory tracts such as; a) beating cilia, b) mucus, c) macrophages and d) underlying respiratory disease [103]. In addition, there are different genes and transporters observed in the respiratory system than the vessels and these two factors interact with the transportation of the eluting drug [104]. The defense mechanisms of the respiratory system are responsible in many situations for the displacement of the stent with catastrophic results in certain cases. The displacement of a stent can cause severe hypoxia and asphyxiation, while this does not happen in the case of coronary heart disease if a stent is blocked. Also, stents are known to be colonized from bacteria biofilm and this is a major reason for replacement. Finally, we have the ability to replace stents, while in the case of PCI this is not an option therefore we expect different results with the prolonged local mTOR local therapy. The mTOR pathway is a future target that could inhibit locally in the airways, since it is responsible for local cell proliferation control and neovascularization [13,26]. In the study by Schlomi et al. [105] the protective role of immunosuppression prolonged the granuloma tissue formation in BMS. The patients in the study received cortisone derivatives, mycophenolatemofetil and tacrolimus a macrolide. We have data regarding the drug eluting stents (DES) of first, second and third generation [33]. These informations could assist in identifying the proper stent for experimentation in the airways as an anti-proliferative local treatment against abnormal granuloma tissue formation. We have indications that the DES are responsible for early vessel wound healing [106,107]. However; there are some contradictory data where SES stents inhibited restenosis when they were placed proximal to a BMS stent [69], but when two SES stents were placed closely they formed a membranous diaphragm formation [70]. Therefore there are still factors affecting the local interaction of the eluting drug with the tissue. Metformin has been identified to impair the vascular endothelial recovery [108]. Young age and insulin dependent diabetics are also factors affecting the target lesion vascularization probably because young age is associated with increased cell proliferation and insulin is considered a growth hormone [59]. There has been also reported the case were zotarolimus eluting stents induced hypersensitivity pneumonia, however; in this case it was not clarified whether this incidence occurred due to the zotarolimus or the polymer coating [51]. The polymer coating has been reported to induce hypersensitivity pneumonitis [40]. Based on the RADAR report further investigation in polymer free coating has been initiated and currently polymer-free phospholipid encapsulated sirolimus stents are being investigated [28]. Moreover; it has been observed that stem cell are mobilized in the wound site but when Granulocyte colony-stimulating factor (G-CSF) was administered in patients with SES implanted stents the endothelial dysfunction was attenuated [109]. Based on the published data we consider the effectiveness of the stents within the following order zotar olimus>everolimus>sirolimus> paclitaxel. Paclitaxel eluting stents have evaluated for minimal invasive emphysema treatment in patients eligible according to the NETT trial [110-112]. Results from the first studies in animals and patients indicate that the stents have prolonged patency the shorter time being 18 weeks and the adverse effects are restricted to the technique of the stent placement [113-116]. However; it has to be stated that these studies evaluated the clinical efficiency of the stents and not their pharmacological properties and local tissue-stent interaction. There are no data regarding the depth of drug penetration and local adverse effects such as the formation of granuloma tissue. A further evaluation of these patients should elicit partially the pharmacodynamic interaction of the paclitaxel eluting stent with the local tissue. Moreover these stents were very short in length and their application was traumatic therefore again no clear conclusions can be drawn. The traumatic insertion although minimal still is a reason for local stress and inflammation, which subsequently enhance the granuloma tissue formation. Another drug which has recently applied as a stent coating is docycycline which. It was observed that doxycycline significantly lowered the matrix metalloproteinase-9 (MMP-9) concentrations and bacterial colonization locally and enhanced the local healing in sinus surgery [117]. Therefore we should consider broadening the spectrum of the drugs that we can as stent coating, based on the concept that different drugs have different behavior in the target tissue independently of the local released concentration [102,118,119]. The group by Chao et al. [120] investigated cisplatin eluting stents for airways malignancy in rabbits. Moreover, the group by Zhu et al. [121] evaluated mitomycin C eluting stents (bioabsorbable and silicone) again in rabbits. Restenosis was observed in only half of the rabbits with bioabsorbable stents after 12 weeks in comparison to the silicone stent group. These studies present new drug designing techniques for local therapy. Currently we are evaluating in a 3D airway constructed from fibroblasts and the ability of sirolimus to control cell proliferation. Another group investigated the minimum inhibitory dosage of paclitaxel in human fibroblasts [122]. It is more likely that the first stents to be evaluated as a local anti-proliferative treatment would be sirolimus stents. The different layers of the airways and depth of drug penetration are major factors affecting the abnormal cell proliferation. There are the factors of local pressure which induces stress and inflammation and the bacteria biofilm which also induces abnormal neovascularization and inflammation. In any case we expect that local anti-inflammatory and immunomodulatory treatment coating the implanted stents to prolong safe airway passage, at least in the first stage of development.

## Figures and Tables

**Table 1:** Trials using eluting stents.

Drug	Methods	Results	Study	Ref
EES vs SES	Percutaneous Coronary Intervention	Less stent thrombosis, reduction in the risk for myocardial infarction and repeat revascularization	Review	[71]
EES second generation vs. SES 643 patients	Small vessels <2.5mm	9.1% major cardiovascular events in EES and 8.6% for SES, 0% thrombosis for EES and 1.2% for SES	Retrospective (1 year follow up)	[72]
EES vs. FG-SES and EES vs. FG-PES 2.126 patients	Percutaneous Coronary Intervention	Lower TVR, less ST and a trend towards lower MACE	2 year follow-up (GHOST)	[75]
ABPB stents vs. DPES stents 2.707 patients	Percutaneous Coronary Intervention	ABPB stents are as safe and efficacious as the current standard of a DPES stents with a durable biocompatible polymer	12 month follow-up (COMPARE II) Randomized	[77]
EES vs. SES 207 patients	Coronary Total occlusion	EES less MACE, and less restenosis	12 month follow-up Randomized	[73]
EES vs. SES 278 patients	*de novo* coronary arterial lesions	EES less plaque volume index, relative change index, less late acquired stent malapposition and positive peri-stent vascular remodeling	9-month follow-up (EXCELLENT)	[74]
EES vs. FG-SES 317 patients	Saphenous vein graft lesions	EES less TLR, TVR, ST and MACE	2 year follow-up randomized	[76]
ZES vs. EES 5.054 patients	Percutaneous Coronary Intervention	Comparable safety and efficacy (even in off label patients)	1 year follow-up (Excellent-Resolute)	[78]
ZES vs. EES 60 patients	Coronary intervention	ZES rapid neointimal healing compared to EES, however; EES better vascular healing profile at 12 month compared to ZES	3 and 12 month follow-up	[68]
ZES vs. EES 1.391 patients	Percutaneous Coronary Intervention	Comparable safety and efficacy (even in off label patients)	Head-to-head comparison of 2 year follow-up (Twente trial)	[79]
ZES vs. PES 400 patients	Percutaneous Coronary Intervention	Lower revascularization rate for ZES patients	12 month follow-up Randomized 1:1 ratio	[81]
ZES vs. SES vs. EES 225 patients	Bifurcation Percutaneous Coronary Intervention	ZES improves performance and “side-branch” trouble	225 patients Patients were treated firstly with SES or EES and Afterwards with ZES	[82]
SES vs. ZES I phase 51 patients SES and 46 ZS and II phase 103 patients SES and 104 patients ZES	Total coronary occlusion	Comparable results for Resolute ZES and SES, Superior results for Endeavor ZES vs. SES	8 month follow-up	[83]
Endeavor ZES, FG-DES and BMS 3.616 patients	Percutaneous Coronary revascularization	ZES lower TLR, but similar to FG-DES, lower MACE with ZES in 5 year follow-up	5 year rates	[84]
PES, BMS, PTA	Superficial femoral arterial lesions	Long term superiority of PES to PTA and BMS	2 year evaluation, randomized controlled trial	[85]
SES, PES and BMS 420 patients	Intracoronary stenting	CYPHER and TAXUS had lower angiographic restenosis and late loss than BMS	November 1995 to June 2011	[86]
PES vs. SES 632 consecutive patients	Percutaneous Coronary Intervention	MACE equally compared and the stent type was not a predictive factor for MACE and TLR	6 year matched cohort study	[88]
PES vs. SES 127 patients	Primary Percutaneous Coronary Intervention	No statistical differences in MACE and ST in the 2 year follow-up	2 year follow-up	[87]
PES vs. EES 770 patients	Left main coronary artery	Comparable safety and efficacy for a 3 year follow-up	3 year (ESTROFA-LM)	[23]
Coroflex Please and TAXUS, 945 patients	Percutaneous Coronary Intervention	Coroflex was inferior to TAXUS, based on clinical and angiographic findings	9 month, prospective, open-label, randomized, controlled study	[89]
Bivalirubin and PES, 3329 patients	Percutaneous Coronary Intervention	LAD PCI patients had higher MACE adverse events in comparison to non-LAD PCI	3 year follow-up (HORIZON-AMI trial)	[90]
SES, 611 patients	SES-associated ST	Abnormal angiographic findings such as; stent fracture and peri-stent contrast staining were found in the very late stent thrombosis Patient group	12 month follow-up (RESTART) Japanese registry	[57]
BMS vs. SES, 200 patients	Total Coronary Occlusions	Superior results for SES patients with in-stent VLLL compared to BMS *p*=0.09, comparable results for in-segment VLLL *p*=0.89	5-year follow-up, (PRISON II) study	[58]
SES vs. EES, 571 patients	Percutaneous Coronary Intervention	Stent fracture and peri-stent contrast staining were lower in the EES group *p*=0.18	12 month follow-up, (RESET) trial	[65]
SES, 249 patients	Percutaneous Coronary Intervention	No association between late TLR and lesion characteristics. Factors affecting TLR were insulin treated diabetes mellitus and young age	5 year follow-up	[59]
SES, 249 patients	Percutaneous Coronary Intervention	The clinical SYNTAX score predicts long-term outcomes among SES-treated patients better than the SYNTAX score	5 year follow-up	[64]
SES vs. BMS, 48 patients	Percutaneous Coronary Intervention	SES stents induce vasoconstriction and endothelial dysfunction	6 month follow-up, randomized	[67]
BMS vs. SES, 115 patients	Percutaneous Coronary Intervention	SES implantation close to BMS inhibits neointimal proliferation in the BMS	retrospective	[69]
SES vs. BMS, 434 patients	Percutaneous Coronary Intervention	MACE events were the same for both groups after seven years and TLR was increased in SES group after 7 years in comparison to BMS	7 year follow-up	[80]
FG-DES vs. BMS	Percutaneous Coronary Intervention for STEMI	SES and PES significant reduction in TVR *p*<0.0001	Median range 1.095 days	[60]
SES vs. ES vs. PES, 1481 patients	Acute coronary syndrome	EES presented lower long term MACE rate in comparison to SES and PES	2 year follow-up	[66]
PLLA-SES vs. BMS	Left coronary ostium PLLA placement	1 out of 12 stents had 50%stenosis	Animal study	[30]
Polymer-free phospholipid encapsulated-SES	Dose-finding study	Reduced neointimal proliferation and low systemic drug release	Animal study	[28]
Abluminal groove-filled biodegradable polymer-SES vs. EES 458 patients	*de novo* coronary lesions	No definite or probable stent thrombosis was observed in both groups	12 month follow-up (TARGET I) trial	[27]
Biolimus-eluting biodegradable polymer-coated stent vs. durable polymer-coated SES, 2468 patients	Percutaneous Coronary Intervention	Inferiority of Biolimus-eluting biodegradable polymer-coated stent-SES	12 months follow-up, (SORT OUT V)	[56]
Nanoporous cell-specific pharmacokinetic eluting stent vs. SES vs. BMS	Porcine coronary model	Reduced neointimal formation and increased reendothelialization	*in vitro, in vivo*	[29]
SES	Review	Review	Review	[33]

**SES;** sirolimus eluting stents, **EES;** everolimus eluting stents, **ZES;** zotarolimus eluting stents, **PES;** paclitaxel eluting stents, **BMS;** Bare Metal Stents, **ST;** stent thrombosis, **FG-SES;** First-Generation-Sirolimus Eluting Stents, **ABPB;**abluminal biodegradable polymer biolimus-eluting stent, **DPES;** durable polymer everolimus-eluting stent, **TLR;** target lesion revascularization, **MACE;** major cardiac adverse evants (death, myocardial infarction), **TVR;** ischemia-driven target vessel revascularization, **FG-DES;** First Generatio-Drug Eluting Stents; **LAD PCI;** left anterior descending artery undergoing percutaneous coronary intervention, **STEMI;** ST segment elevation myocardial infarction, **PTA;** percutaneous transluminal angioplasty, **PLLA;** paclitaxel-coated poly-L-lactide acid biodegradable biopolymer stent.

## Abbreviations

SES: Sirolimus Eluting Stents
EES: Everolimus Eluting Stents
ZES: Zotarolimus Eluting Stents
PES: Paclitaxel Eluting Stents
BMS: Bare Metal Stents
ST: Stent Thrombosis
STEMI: ST-Segment Elevation Myocardial Infarction
TVR: Target Vessel Revascularization
TLR: Target Lesion Vascularization
PCI: Percutaneous Coronary Intervention
MACE: Death Events and Myocardial Infarction Events
RADAR project: Research on Adverse Drug Events and Reports
PLLA: Paclitaxel-coated poly-L-lactide acid
MMP-9: Matrix Metalloproteinase-9
LAD: Left anterior Descending Artery
CAPTAIN: Cardiovascular Atherosclerosis and Percutaneous Transluminal Interventions
PTA: Percutaneous Transluminal Angioplasty
EST: Early-ST events
LST: late-ST events
VLST: Very late-ST events
FG-DES: First-Generation-Drug Eluting Stents
CSS: Clinical SYNTAX
VLLL: Very Late Luminal Loss
ALLL: Additional Late Luminal Loss
CREG: Cell-Specific Pharmacokinetic Effect Stent
IVUS: Intravascular Ultrasound
LASM: Late Acquired Stent Malapposition
SG-EES: Second Generation-Everolimus Eluting Stents
